# Quantitative proteomics analysis of early recurrence/metastasis of huge hepatocellular carcinoma following radical resection

**DOI:** 10.1186/1477-5956-12-22

**Published:** 2014-05-01

**Authors:** Xinhui Huang, Yongyi Zeng, Xiaohua Xing, Jinhua Zeng, Yunzhen Gao, Zhixiong Cai, Bo Xu, Xiaolong Liu, Aimin Huang, Jingfeng Liu

**Affiliations:** 1 Mengchao Hepatobiliary Hospital of Fujian Medical University, 350025 Fuzhou, People’s Republic of China; 2 The Liver Center of Fujian Province, Fujian Medical University, 350025 Fuzhou, People’s Republic of China; 3 Liver Disease Center, The First Affiliated Hospital of Fujian Medical University, 350005 Fuzhou, People’s Republic of China; 4 Department of Pathology, School of Basic Medical Science, Fujian Medical University, 350004 Fuzhou, People’s Republic of China

**Keywords:** Huge hepatocellular carcinoma (H-HCC), Early recurrence/metastasis, Quantitative proteomics, iTRAQ, Potential prognostic biomarker

## Abstract

**Background:**

Hepatic resection is the preferred treatment for huge hepatocellular carcinoma (>10 cm in diameter; H-HCC). However, the patients with H-HCC suffer from poor prognosis due to the early recurrence/metastasis. The underlying mechanism of H-HCC’s early recurrence/metastasis is currently not well understood.

**Results:**

Here, we describe an Isobaric Tags for relative and absolute quantification (iTRAQ)-based quantitative proteomics approach to analyze the early recurrence/metastasis related proteins of H-HCC after radical resection through multidimensional chromatography coupled with tandem mass spectrometry (2DLC-MS/MS). The different protein expression profiles between the early recurrence/metastasis within 6 months(R/M_≤6months_) and late recurrence/metastasis within 6–12 months after surgery (R/M_6-12months_) were confirmed and might reveal different underlying molecular mechanisms. We identified 44 and 49 significantly differentially expressed proteins in the R/M_≤6months_ group and the R/M_6-12months_ group compared to the group who had no recurrence within 2 years post surgery (the NR/M group), respectively. Moreover, among those proteins, S100A12 and AMACR were down regulated in the R/M_≤6months_ group but up-regulated in the R/M_6-12months_ group; and this regulation was further confirmed in mRNA and protein level by Q-PCR, Western-Blot and Immunohistochemistry (IHC).

**Conclusions:**

This current study presents the first proteomic profile of the early recurrence/metastasis of H-HCC. The results suggest that S100A12 and AMACR might be potential prognostic markers for predicting the early recurrence/metastasis of H-HCC after hepatectomy.

## Background

Hepatocellular carcinoma (HCC), the fifth most common cancer worldwide [1], is reported to be the second leading cause of cancer death in China [2]. Huge hepatocellular carcinoma (H-HCC) with the feature of diameter larger than 10 cm, is a special subtype of HCC. Despite the proven feasibility and safety of surgical resection for H-HCC [3,4], the prognosis of H-HCC patients remains poor, mainly due to the intra-hepatic recurrence and/or extra-hepatic metastasis. The incidence of recurrence/metastasis within 12 months in the residual liver of H-HCC patients who underwent curative resection, ranges from 50% to 70% [5-7]; and the median survival after recurrence/metastasis is only 13 months [8]. This has largely blocked the curative efficiency and long-term survival of hepatectomy.

In the last two decades, various molecular alterations have been found to correlate with early recurrence/metastasis of HCC [9-12]. However, the detailed underlying molecular mechanisms of the early recurrence/metastasis of H-HCC are still not well understood. The recurrence/metastasis of HCC is a complicated process, which is resulting from combined effects of multiple factors [13].

Studies focusing on individual gene or protein might be insufficient for elucidating the biological natures of the malignant behavior of tumor. Quantitative proteomics approaches, which are able to give an overview of the global protein profile alternation under pathological conditions, have been proposed to be extremely useful tools in studying the recurrence/metastasis behavior of cancer [14-18]. Several groups have been extensively applying the proteomics approach to elucidating the biological behaviors of HCC; Sun et al. have studied the differentially expressed proteins in tumor and adjacent non-tumor tissue samples, and found that Hcp70/Hsp90-organizing protein and heterogeneous nuclear ribonucleoproteins C1/C2 could be potential biomarkers in HCC [19]; Orimo et al. have studied the protein expression alternations in 45 surgically resected tissues with different degree of histological differentiation, and identified APC-binding protein EB1 (EB1) as a potential prognostic biomarker for HCC [20]. However, the traditional proteomics based approaches suffer from low throughput non-quantitative information coupled with difficulties in separating and/or detecting low abundant proteins [21], post translationally modified proteins [22,23], as well as those proteins with a pI value lower than 4 or higher than 9 [24].

Recently, high-throughput quantitative proteomic techniques have been developed [25-28]. In particular, isobaric tags for relative and absolute quantification (iTRAQ) labeling followed by nano liquid chromatography-mass spectrometry (NanoLC-MS/MS) is an extremely effective method for simultaneous quantitative comparison and analyzing the protein expression profile of multiple samples. Especially, this has been useful for studying disease associated and low abundance proteins [29-34] with good sensitivity. So far, several groups have reported the applying of iTRAQ based quantitative proteomics approach in the study of hepatic cancer, especially for the screening of diagnostic or prognostic protein biomarkers. He et al. have reported the serum biomarker screening of AFP negative HBV related HCC through the iTRAQ based approach [35]; Ko et al. have reported the iTRAQ based quantitative analysis of HCC cancer stem cell proteome [36]; Huang et al. have reported the iTRAQ based serum biomarker screening of the HCC micro-vascular invasion [37]; Qin et al., Yu et al. and Wang et al. have reported the screening of metastatic related proteins of HCC through iTRAQ based approach [33,38,39]; the iTRAQ based quantitative study of proteome change during HBV infection has also been reported [40,41]. However, the application of iTRAQ labeling in studying the molecular mechanisms and screening for biomarkers associated with the early recurrence/metastasis of H-HCC has not yet been reported to our knowledge.

In the present study, we applied the iTRAQ based quantitative proteomic approach (iTRAQ-2DLC-MS/MS) to quantitatively analyze the protein profiles and alternations of the early recurrence/metastasis in H-HCC after radical hepatic resection, and tried to identify the potential prognostic markers and reveal the underlying mechanism of the early recurrence/metastasis in H-HCC.

## Results

### The quantitative proteomics of the recurrence/metastasis of H-HCC

Here, the proteins were identified and quantified according to the following criteria: a protein is reported if a quantification ratio was obtained using at least 2 unique peptides with “unused” confidence cutoff (PortScore) >1.3 (95%); 1408 proteins (1818 proteins before grouping) were identified from 23767 distinct peptides; and 1279 unique proteins were quantified from samples analyzed on QSTAR Elite. A complete list of proteins identified in our study is shown in Additional file 1; the details of proteomic analysis including sequence coverage, score of unique peptides, quantification results with percentage variability are included in the list as well. The protein expression alternations in different groups were calculated from the intensity ratio of the iTRAQ reporter ions from the derived peptides. Full details of the dataset and supplementary can be accessed through ftp://epigenome.fudan.edu.cn/ by using FlashFXP.

### Categorization and functional annotation of proteins quantitated in R/M_≤6months_ and R/M_6-12months_

Functional annotations of all the identified proteins through IPA are shown in Additional file 2: Figure S1. Categories are based on primary localization, which include nucleus (17%), extracellular space (9%), cytoplasm (65%), plasma membrane (6%), while 3% of the identified proteins is currently not clearly classified (Additional file 2: Figure S1A). Moreover, molecular type of proteins was also analyzed, of which nearly half of the identified proteins are enzymes and transporters (Additional file 2: Figure S1B).

At the same time, functional annotation of all the identified proteins by Gene Ontology (GO) including biological process, sub-cellular localization and molecular function are shown in Additional file 2: Figure S2 and the top 10 involved functions were ranked in term of the enrichment.

**Figure 1:**
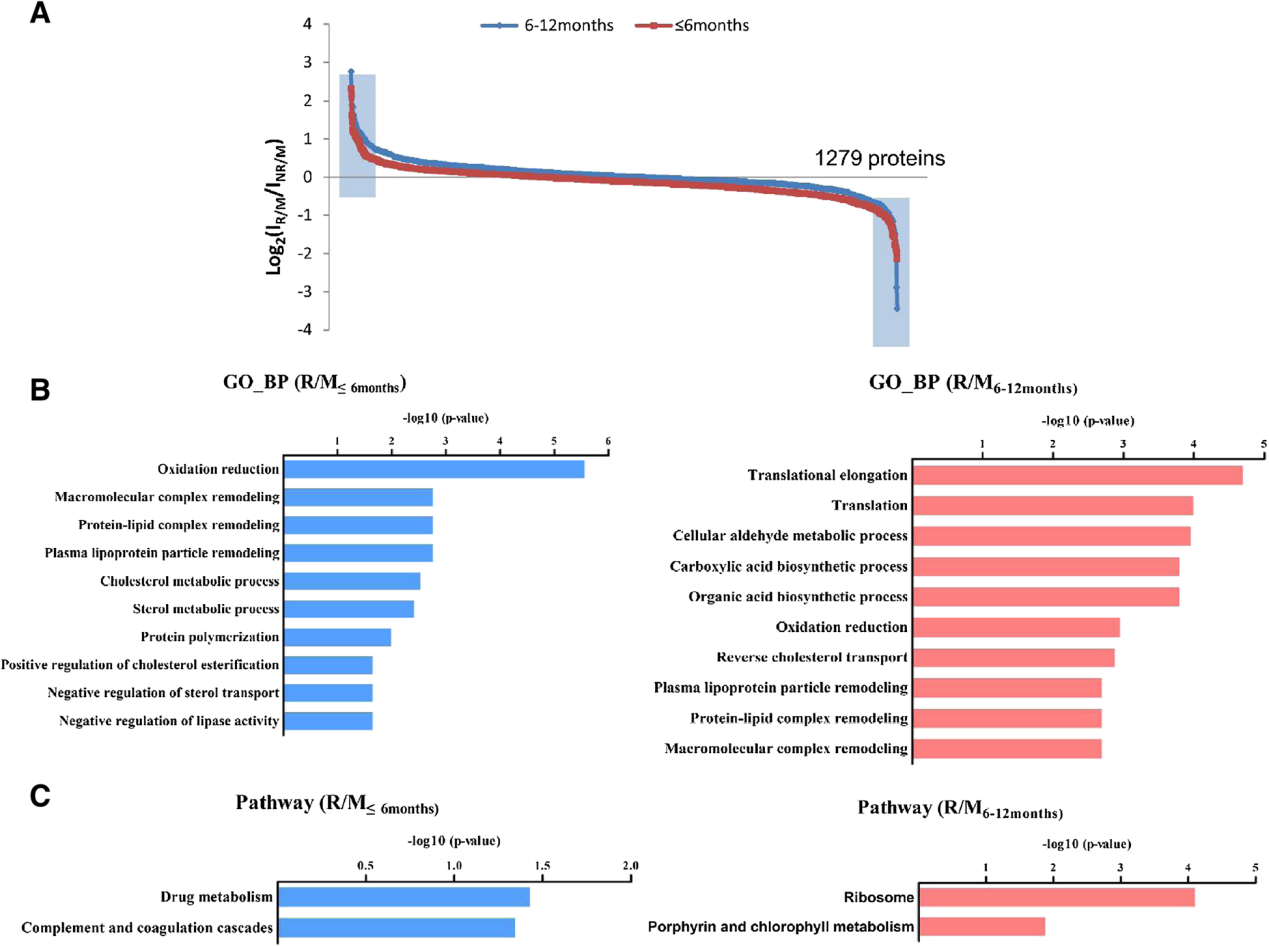
**The sub-cellular distribution and biological functional annotation of identified proteins. (A)** The protein expression distributions of R/M_≤6months_ and R/M_6-12months_; the differential expressed proteins in R/M_≤6months_ and R/M_6-12months_ comparing to the NR/M group was indicated by the blue tag. **(B)** Go analysis for biological functions of the differentially expressed proteins in R/M_≤6months_ and R/M_6-12months_, the top 10 were shown in panel B. **(C)** KEGG pathway enrichment analysis of the differentially expressed proteins in R/M_≤6months_ and R/M_6-12months_ group (p < 0.1).

In this study, we designated the differentially expressed proteins with the fold change cutoff ratio < 0.5 or >2.0. Figure 1A shows iTRAQ fold changes of all identified proteins, and a small subset of differential expressed proteins in both R/M_≤6months_ and R/M_6-12months_ group compared to the NR/M group (indicated by the blue tag). 46 differentially expressed proteins (15 up-regulated and 31 down-regulated) were detected in the R/M_≤6months_ group and 49 differentially expressed proteins (33 up-regulated and 16 down-regulated) were detected in the R/M_6-12months_ group upon comparison with the NR/M group (Additional file 1: Table S1 and Table S2). Functional annotations of these differentially expressed proteins (compared to the NR/M group) were analyzed as follows: (A) Gene Ontology (GO) analysis to reveal the different involved biological functions in R/M_≤6months_ and R/M_6-12months_ (Figure 1B), (B) KEGG pathway enrichment analysis which reveals that the drug metabolism as well as complement and coagulation cascades were mainly involved in R/M_≤6months_; metabolism related signaling pathways (like ribosome metabolism, porphyrin and chlorophyll metabolism) were actively involved in the R/M_6-12months_ group (Figure 1C) and (C) GO analysis of cell components and molecular function analysis to reveal the different characters of the early (R/M_≤6months_ group) and late (R/M_6-12months_ group) recurrence/metastasis patients (Additional file 2: Figure S3). All the differentially expressed proteins in R/M_≤6months_ and R/M_6-12months_ comparing to the NR/M group have been listed in Additional file 1: Table S1 and Table S2, respectively. Comparison of results from Additional file 1: Table S1 and Table S2, clearly reveals that over 80% of the protein expression alternations were different between R/M_≤6months_ group and R/M_6-12months_ group. It indicates that the early recurrence/metastasis might be induced by different factors compared to the late stage recurrence/metastasis; indicating that they are possibly undergoing different molecular mechanisms.

### Differentially expressed proteins in R/M_≤6months_ and R/M_6-12months_

**Figure 2:**
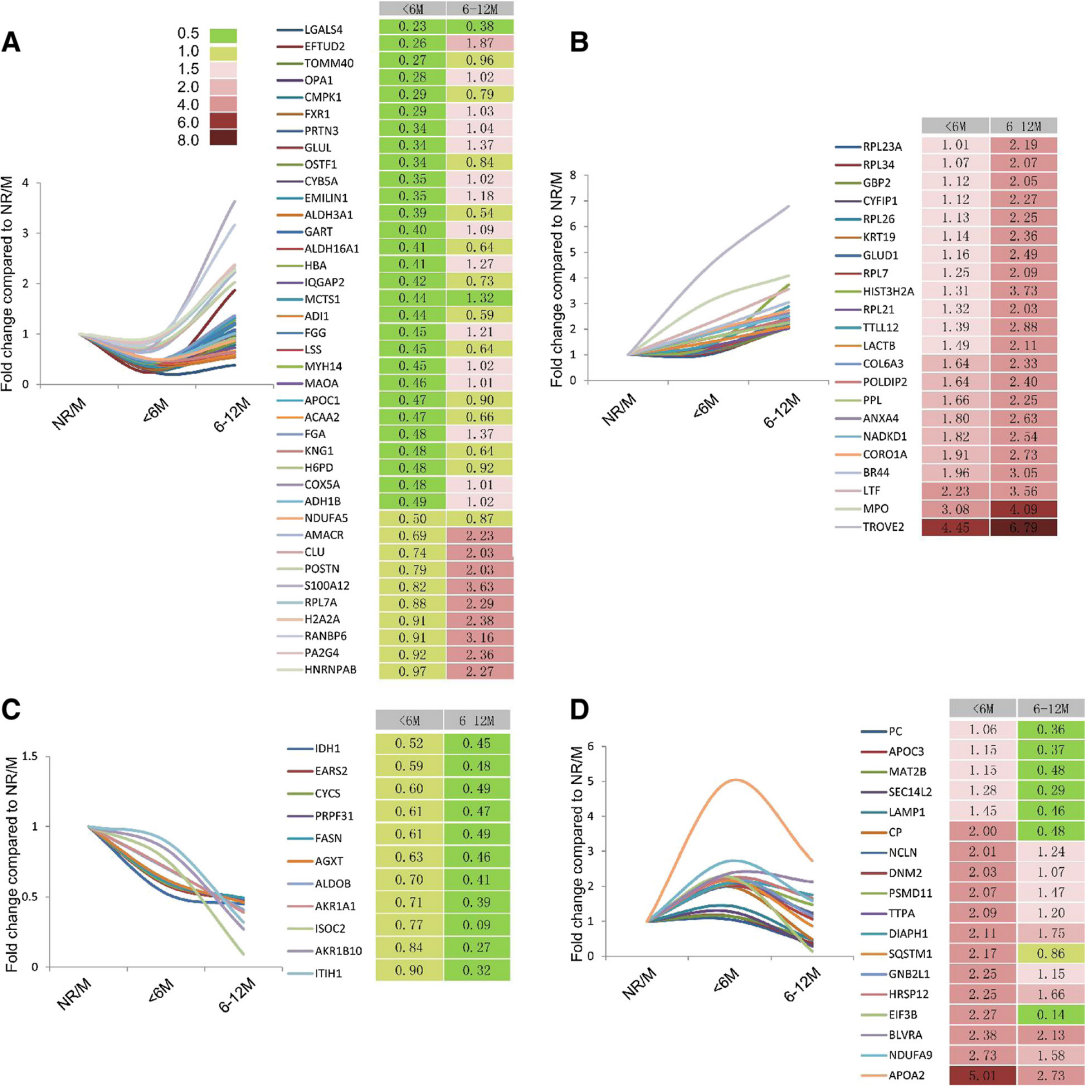
**Alternation tendency of proteins in R/M**_**≤6months **_**group and R/M**_**6-12months **_**group comparing to the NR/M group. (A)** The proteins which are decreased in the R/M_≤6months_ group, but increased in the R/M_6-12months_ group comparing to the NR/M group; these proteins are mostly related with glycolysis and protein synthesis. **(B)** The proteins which are highly expressed in both R/M_≤6months_ group and R/M_6-12months_ group comparing to the NR/M group; these proteins are mostly involved in protein synthesis process. **(C)** The proteins which are lowly expressed in both R/M_≤6months_ group and R/M_6-12months_ group comparing to the NR/M group; these proteins are mostly related to the amino acid metabolic process. **(D)** The proteins which are increased in the R/M_≤6months_ group, but decreased in the R/M_6-12months_ group comparing to the NR/M group; these proteins are involved in multiple signaling pathways.

By carefully comparing the protein expression alternations, there are 4 types of trends according to the protein fold changes. The first type is the altered proteins decreased in the R/M_≤6months_ group, but increased in the R/M_6-12months_ group comparing to the NR/M control group_;_ in these proteins, the enzymes related to the glycolysis (ADH1B, ALDH3A1, MAOA, ALDH16A1) and the proteins related to protein synthesis (RPL7A, PA2G4, MCTS1, EFTUD2) were involved in (Figure 2A). While ribosome proteins related to protein synthesis were found highly expressed in both R/M_≤6months_ group and R/M_6-12months_ group comparing to the NR/M group, such as RPL7, RPL26, RPL21, RPL34, RPL7, RPL23A etc. (Figure 2B). Figure 2C shows proteins decreased in both R/M_≤6months_ group and R/M_6-12months_ group compared to the NR/M control group, which are involved in the amino acid metabolic process (EARS2, IDH1, PASN, AGXT). Lastly, there is exists another type of differentially expressed proteins whose expression is increased in the R/M_≤6months_ group, but decreased in the R/M_6-12months_ group in comparison with the NR/M group and lying scattered in multiple signaling pathways (Figure 2D).

By carefully comparing the protein expression alternations, we filtered 8 proteins that altered their expression in both R/M_≤6months_ group and R/M_6-12months_ group relative to the NR/M group, which might closely relate to the metastatic propensity (Table 1). These proteins mostly belong to the secretory proteins category (Galectin-4 and S100A12), transport proteins (Lactotransferrin and Apo-lipoprotein A-II), transcription and translation factors (60-kDa-SS-A/Ro ribonucleoprotein and Eukaryotic translation initiation factor 3 subunit B, eIF3b), and antioxidant proteins (Biliverdin Reductase A and Alpha-methy-lacyl-CoA racemase, AMACR). Although these proteins have rarely been linked with cancer, the signaling pathways regulated by these proteins and their protein families play an important role in oncogenesis, and are extensively involved in the invasion and metastasis of tumor [42-49].

**Table 1:** **List of common proteins altered their expression in both R/M**_**≤6months **_**group and R/M**_**6-12months **_**group**

**Accession number**	**%Cov**	**Protein name**	**iTRAQ ratio 117:121(R/M**_**≤6months**_**)**	**iTRAQ ratio 119:121(R/M**_**6-12months**_**)**
LEG4_HUMAN	33.1	Galectin-4	0.2291	0.3837
S10AC_HUMAN	27.2	Protein S100-A12	0.3052	3.6308
AMACR_HUMAN	42.9	Alpha-methylacyl-CoA racemase	0.3585	2.2284
TRFL_HUMAN	18.6	Lactotransferrin	2.2284	3.5645
EIF3B_HUMAN	13.1	Eukaryotic translation initiation factor 3 subunit B	2.2699	2.1355
BIEA_HUMAN	17.6	Biliverdin reductase A	2.3768	2.1281
RO60_HUMAN	13.9	60 kDa SS-A/Ro ribonucleoprotein	4.4463	6.7920
APOA2_HUMAN	73	Apolipoprotein A-II	5.0119	2.729

In these proteins, we could clearly see that the protein S100A12 and AMACR show different alternation tendency between R/M_≤6months_ group and R/M_6-12months_ group; the protein S100A12, which has been reported to regulate the calcium metabolism and arachidonic acid metabolism in the neutrophil [50], is down regulated more than 3.3 folds in R/M_≤6months_ group compared to the NR/M group, but up regulated more than 3.6 folds in R/M_6-12months_ group relative to the NR/M group. AMACR, which has been reported to regulate the metabolism of fatty acid, cholalic acid [51] and closely associated with the differential diagnosis of prostatic cancer [52], was down regulated by over 2.8 folds in R/M_≤6months_ group compared to the NR/M group, but up-regulated more than 2.2 folds in R/M_6-12months_ group compared to the NR/M group. Since these two proteins have clearly different expression profiles in the R/M_≤6months_ group and the R/M_6-12months_ group, they might be interesting potential biomarkers for predicting early recurrence/metastasis after radical resection in H-HCC.

### Functional analysis of the protein expression alternations in the recurrence/metastasis of H-HCC

To analysis the roles of the protein expression alternations in the recurrence/metastasis of H-HCC, we used the IPA software to study the key signaling pathways and networks of those differentially expressed proteins. The analyzed results suggest that different signaling pathways are indeed involved in the early (R/M_≤6months_ group) and late (R/M_6-12months_ group) recurrence/metastasis patients, although there are common signaling pathways involved in as well. The IPA software identified that the altered protein expressions in the R/M_≤6months_ group patients are mostly involved in ERK1/2 signaling pathway and TNF signaling pathway, but the altered protein expressions in the R/M_6-12months_ group patients are mostly involved in TNF signaling pathway and NFκB signaling pathway.

**Figure 3:**
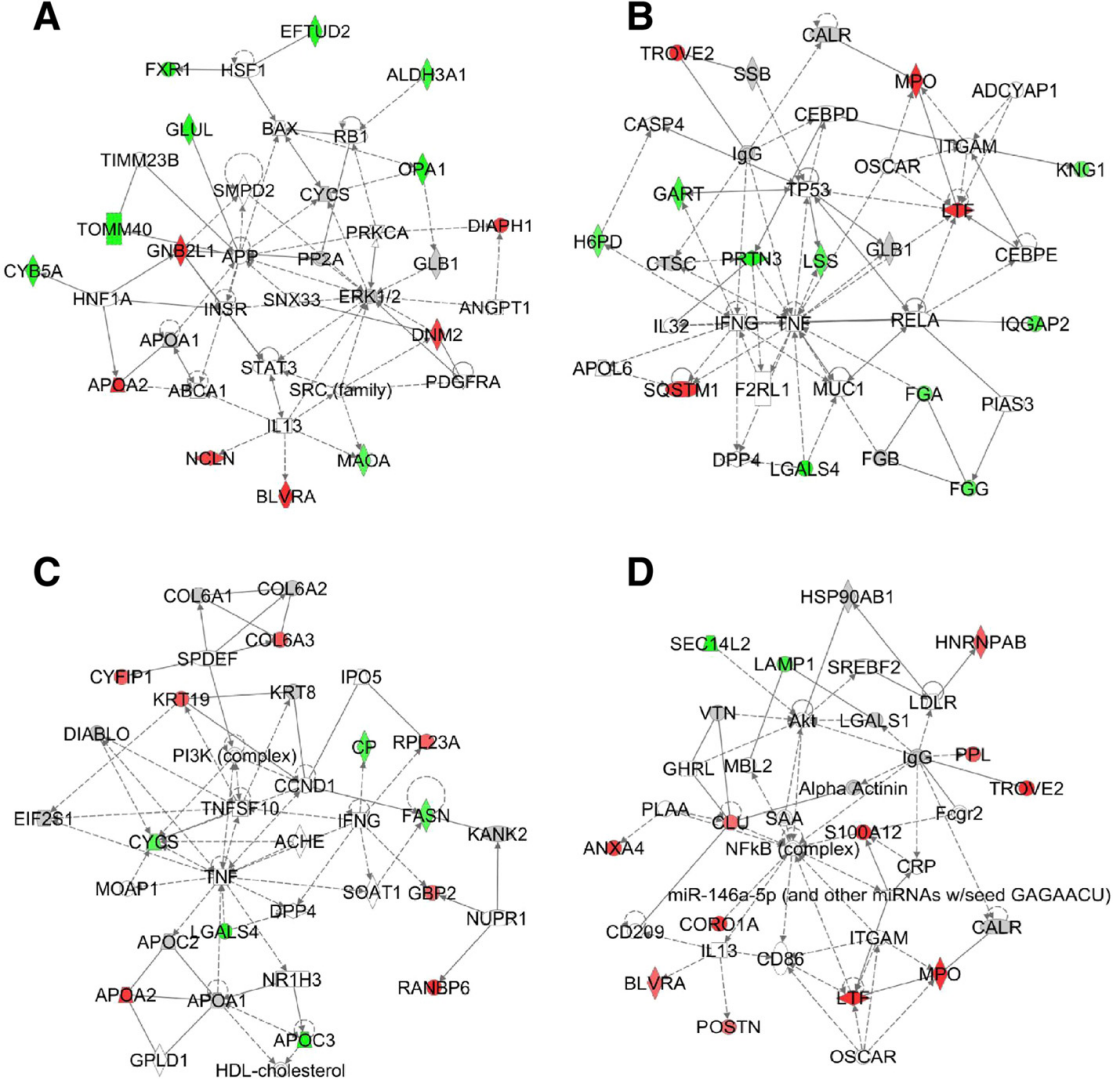
**The key signaling pathways involved in the early recurrence/metastasis (R/M**_**≤6months**_**) and late recurrence/metastasis (R/M**_**6-12months**_**) of H-HCC. (A)** The alternations of the ERK1/2 signaling pathway in the R/M_≤6months_ group, including 6 up-regulated proteins (red labeling) and 7 down-regulated (green labeling) proteins. **(B)** The alternations of the TNF signaling pathway in the R/M_≤6months_ group, including 4 up-regulated proteins (red labeling) and 9 down-regulated proteins (green labeling). **(C)** The alternations of the TNF signaling pathway in the R/M_6-12months_ group, including 7 up-regulated proteins (red labeling) and 5 down-regulated proteins (green labeling). **(D)** the alternations of the NFκB signaling pathway in the R/M_6-12months_ group, including 9 up-regulated proteins (red labeling) and 2 down-regulated (green labeling) proteins.

In the R/M_≤6months_ group patients, we could see that 14 proteins (including 6 up-regulated proteins and 8 down-regulated proteins) were involved in the ERK1/2 signaling pathway (Figure 3A); and 13 proteins (including 4 up-regulated proteins and 9 down-regulated proteins) involved in TNF signaling pathway (Figure 3B). In the R/M_6-12months_ group patients, 12 proteins (including 7 up-regulated proteins and 5 down-regulated proteins) involved in the TNF signaling pathway (Figure 3C); and 13 proteins (including 11 up-regulated proteins and 2 down-regulated proteins) involved in the NFκB signaling pathway (Figure 3D). Although, all of these 3 signaling pathways are actively associated with cancers [53-55], as describing above, only the TNF signaling pathway is extensively involved in both the early and late recurrence/metastasis of H-HCC. The reported functions of TNF signaling pathway in carcinogenesis include tumor initiation, tumor cell proliferation, tumor angiogenesis, and enhancing the invasive property of tumor cells [55,56]; therefore, it is not surprising that the TNF signaling pathway is involved in both groups. Interestingly, although both the ERK1/2 signaling pathway and the NFκB signaling pathway are reported to associated with cancer development, during malignant transformation and metastasis [53,54], only the ERK1/2 signaling pathway is enriched in the early (R/M_≤6months_) recurrence/metastasis patients. ERK1/2 is known an extremely important regulator of cell growth and proliferation [54], therefore, the early recurrence/metastasis H-HCC cells probably have stronger growth and proliferation abilities than late recurrence/metastasis H-HCC cells; but the detailed underlying mechanisms should be further studied.

### The verification of S100A12 and AMACR expression alternation

As mentioned above, the protein S100A12 and AMACR were reversely expressed in the R/M_≤6months_ group (early recurrence/metastasis) and the R/M_6-12months_ group (late recurrence/metastasis). Therefore, they might be potential interesting biomarkers to predict the early and late recurrence/metastasis in H-HCC. Here, we took liver sections from H-HCC patients to verify their expression profiles and predicting potentials in a larger scale. Combing with the patient case history, the expression profiles of both S100A12 and AMACR in different groups were confirmed by Q-PCR at the mRNA level and by Western-blot and immunohistochemistry in the protein level.

**Figure 4:**
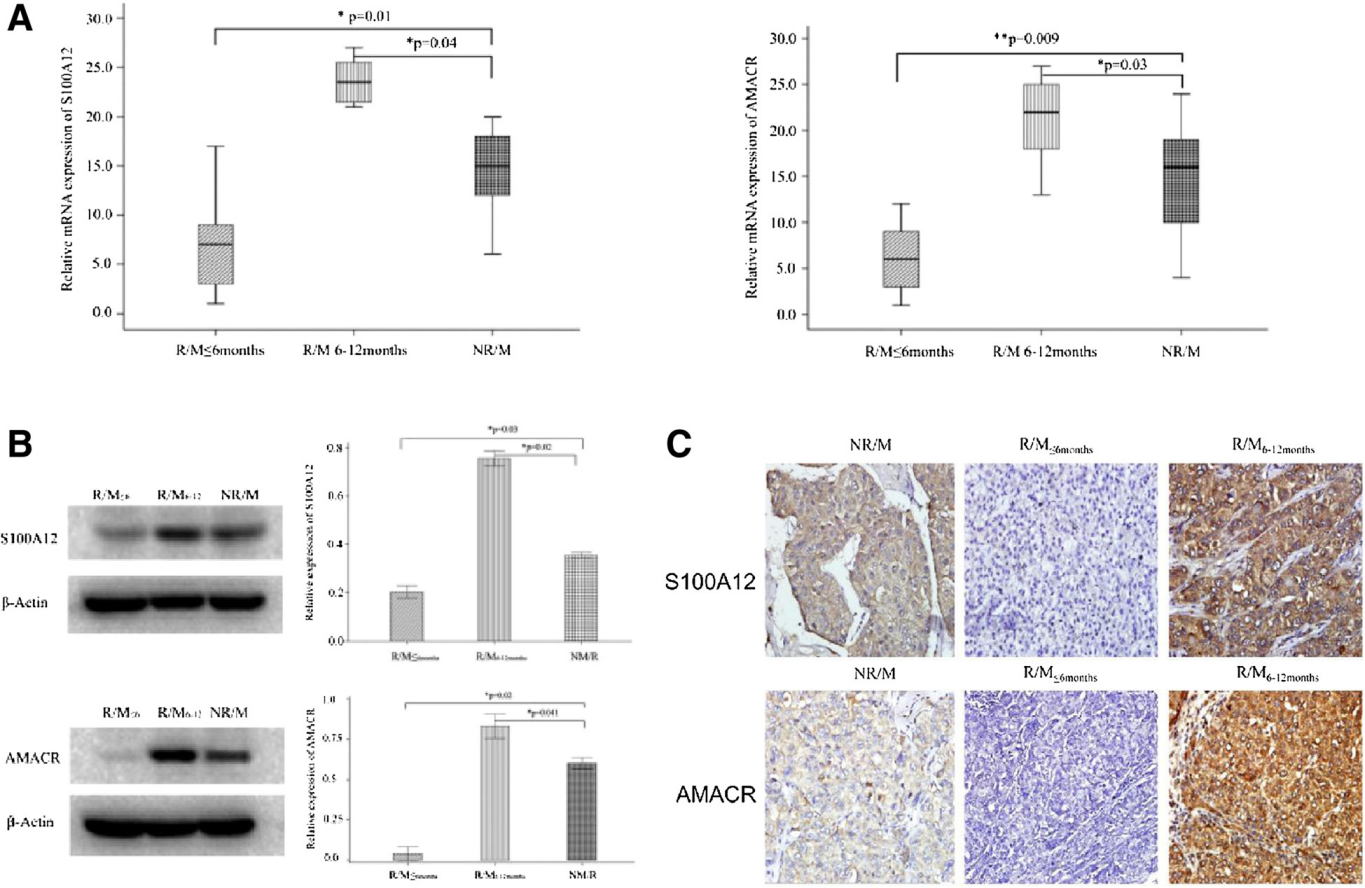
**Verification of differentially expressed proteins in different stage of recurrence/metastasis in huge hepatocellular carcinoma patients. (A)** The mRNA expression level of S100A12 and AMACR (p < 0.05, paired T-test); **(B)** The protein expression level of S100A12 and AMACR when validated by Western-Blot (p < 0.05, paired T-test); **(C)** The immunohistochemistry staining results of S100A12 and AMACR (with 400x magnification). Both S100A12 and AMACR are strongly over-expressed on the patient’s samples from R/M_6-12months_ group, but down regulated on the patient’s samples from R/M_≤6months_ group when it is comparing with the control samples.

As shown in Figure 4A, the gene expression of S100A12 and AMACR were down-regulated 2.5 fold and 14.2 fold in the R/M_≤6months_ group (n = 20 patients), respectively; but up-regulated 5.1 fold and 5.38 fold in R/M_6-12months_ group (n = 20 patients), respectively. As shown in Figure 4B, the protein expression of S100A12 and AMACR are down regulated 1.75 fold and 16.6 fold in the R/M_≤6months_ group (n = 20 patients), respectively; but up-regulated 2.14 fold and 1.38 fold in the R/M_6–12 months_ group (n = 20 patients), respectively. These results thus fitted well with the quantitative proteomics study results.

To further confirm the protein expression and localization profiles of S100A12 and AMACR, the immunohistochemistry was performed on patient’s liver sections. As shown in Figure 4C, both the expression intensity and the positive rate (Tables 2 and 3) of the S100A12 and AMACR were down regulated in the R/M_≤6months_ group but up regulated in the R/M_6-12months_ group, compared to the NR/M group respectively.

**Table 2:** Immunohistochemistry assessment of the S100A12 expression level on patient’s samples with different recurrence/metastasis stage

		**S100A12 expression**			
**Groups**	**Numbers**	**Negative**	**Positive**	**Positive expression rate (%)**	***P values***^**a)**^
R/M_≤6months_ group	20	16	4	20.0	0.048
R/M_6-12months_ group	20	3	17	85.0	0.008
NR/M group	20	9	11	55.0	

^a)^*P* value listed above stands for the significance of the S100A12 positive expression rate when R/M_≤6months_ group and R/M_6-12months_ group compared with NR/M group respectively. *P* value less than 0.05 is taking as significant difference with Chi-square test.

**Table 3:** Immunohistochemistry assessment of the AMACR expression level on patient’s samples with different recurrence/metastasis stage

		**AMACR expression**			
**Groups**	**Numbers**	**Negative**	**Positive**	**Positive expression rate (%)**	***P values***^**a)**^
R/M_≤6months_ group	20	17	3	15.0	0.041
R/M_6-12months_ group	20	2	18	90.0	0.014
NR/M group	20	10	10	40.0	

^a)^*P* value listed above stands for the significance of the AMACR positive expression rate when R/M_≤6months_ group and R/M_6-12months_ group compared with NR/M group respectively. *P* value less than 0.05 is taking as significant difference with Chi-square test.

Combining with the patient’s case history and the expression profiles of S100A12 and AMACR, these two proteins could well distinguish the early (within 6 months) and late (from 6 to 12 months) recurrence/metastasis of H-HCC. They might be potential interesting biomarkers for the predicting of early recurrence/metastasis of H-HCC, but the underlying molecular mechanisms needs to be further dissected.

## Discussions

As reported in our previous clinical studies [57], the early recurrence/metastasis of H-HCC is affected by multiple factors. The independent risk factors for the early recurrence/metastasis occurred within 6 months, and those factors for the late recurrence/metastasis are different and occurred between or after 6 to 12 months. Therefore, the underlying molecular mechanism of the early (occurred within 6 months) and late (occurred from 6 months to 12 months) recurrence/metastasis might be different.

The recent rapid development of proteomic techniques allow a full-scale illustration of the protein expression profile alteration under a particular disease circumstance, and thus in identifying important disease-related protein biomarkers or therapeutic targets [33,35-41]. The HCC associated proteomics is believed to further facilitate the studies on systematic screening of the molecular mechanisms involved in HCC recurrence/metastasis, and in the development of prognosis biomarkers for HCC recurrence/metastasis.

In this paper, we applied the iTRAQ based quantitative proteomics approach to study the overall protein profile alternations in the early and late recurrence/metastasis of H-HCC. Here, we successfully identified 46 and 49 differentially expression proteins in the R/M_≤6months_ group and R/M_6-12months_ group compared to the NR/M group, respectively; however, we fished out only 8 proteins that altered its expression in both R/M_≤6months_ group and R/M_6-12months_ group. In our study, we pooled the samples to standardize the reading since the individual sample is likely to give highly divergent and incoherent results; while with pooled samples one could lose information on individual patient variability and also might mess up the readings if there are sub-groups. However, many of the studies utilizing clinical samples face similar problems, which to certain extent limit the development of clinical proteomics.

Among those identified proteins, over 80% of the expression altered proteins were different between the R/M_≤6months_ group and R/M_6-12months_ group, which suggested different molecular mechanisms operate during the early (within 6 months) and late (from 6 to 12 months) recurrence/metastasis of H-HCC. This finding fitted well with our initial hypothesis. Therefore, those H-HCC patients who had recurrence/metastasis at different time points as per our classification (early or late) might need completely different medical treatment. In such a case, it is necessary to identify novel biomarkers and therapeutic targets for distinguishing, predicting and treating the early and late recurrence/metastasis, which will certainly provide important guidelines for identifying H-HCC patients with high risk of early recurrence/metastasis.

The indentified protein expression alterations involve several biological processes, including carbohydrate metabolism, lipid metabolism, cell-cell communication, apoptosis, proliferation, anti-oxidation, transcription and translation, which are confirming that the malignant behavior of HCC cells is controlled by complicated signaling process. Some of the identified proteins have also been described to be associated with HCC recurrence/metastasis. For example, CK19 is up-regulated 2.35 folds in R/M_6–12 months_ group compared with NR/M group; it has been reported to be highly expressed in HCC tissues, and the patients with positive CK19 results had a higher incidence of pulmonary metastasis within 1 month after receiving surgical resection [58]. Periostin (PN) is a secreted matrix glycoprotein, which has been reported to be closely related with the metastatic potential and prognosis of HCC after surgery [59]; Clusterin has been reported to promote the HCC metastasis via regulating TGF-β1-smad3 signaling and then induction of the epithelial-mesenchymal transition (EMT) process [60]. In our study, the expressions of those proteins have also proved to be altered in the R/M_6-12months_ group patients. Therefore, it is clearly establishes that our quantitative proteomics approach is suitable for studying the recurrence/metastasis of H-HCC.

In our clinical, more than 50% of H-HCC patients have recurrence/metastasis within 6 months after radical surgery [58]. However, we did not have any techniques or biomarkers available to early predict the recurrence/metastasis risky of H-HCC patients so far. Predicting early the recurrence/metastasis potential of the H-HCC patients will improve the prognosis. In our study, we identified that protein S100A12 and AMACR were reversely expressed in the early (within 6 months, down regulated) and late (from 6 to 12 months, up regulated) recurrence/metastasis H-HCC patients; and further validated these findings from clinical samples at the level of both mRNA and protein expression. The recurrence/metastasis of HCC is closely related with clinical parameters such as the size of tumor, tumor capsule, tumor boundaries, portal vein tumor thrombosis (PVTT), intraoperative ascites, cirrhotic nodule, heteroploidy, PCNA (Proliferating Cell Nuclear Antigen) expression and others. Our study shows that the low expression of AMACR is related with the absence of tumor capsule, indistinct tumor boundary as well as presence of portal vein tumor thrombosis (PVTT) and intraoperative ascites, which fits with the earlier recurrence/metastasis of AMACR lower expression group.

S100A12 is a new member of S100 protein family (calcium binding protein family); the gene encoding S100A12 was localized on the human chromosome 1q21 [61], and is normally expressed in neutrophil granulocytes, with low expression in both lymphocytes and monocytes [50]. Its reported function involves calcium regulation and arachidonic acid metabolism in the neutrophile granulocytes [50]; it has also been reported that the expression of S100A12 was up-regulated both at mRNA and protein levels in the patients with colorectal carcinoma relative to healthy volunteers [62]. The AMACR encoding gene is localized on chromosome 5p13 [51]; it is probably involved in the metabolism of fatty acid and cholalic acid [51]. Previous studies have reported the application of AMACR in the diagnosis of prostatic cancer [62,63] and there are also a few studies that reported AMACR could be used as biomarker to distinguish the hepatocellular carcinoma and the benign tissue [64,65]. However, these two proteins were rarely reported to be associated with the prognosis of HCC. In this study, we not only prove that the S100A12 and AMACR were associated with the recurrence/metastasis of H-HCC, but also clearly demonstrate that these two proteins have completely inverse expression profiles between the early (within 6 months, down regulated) and late (from 6 to 12 months, up regulated) recurrence/metastasis of H-HCC, even considering a larger “n” number of clinical samples as employed in this study. Therefore, they might be potentially interesting biomarkers for distinguishing and predicting the early and late recurrence/metastasis of H-HCC. However, the exact molecular mechanisms of how these two proteins involved in the pathogenesis and progression of H-HCC should be further verified and studied. It has been reported that the abnormal expression of S100A12 is closely related with inflammation and vascular invasion of tumor cell [66], over inflammation and vascular invasion are closely related with tumor recurrence/metastasis. Meanwhile, the low expression of AMACR was independently associated with the development of metastasis and lethal in prostate cancer through regulating lipid metabolism and nuclear receptor activity [67]. Whether or not these reported functions are involved in the here reported HCC recurrence/metastasis needs further studies.

Overall, we have used iTRAQ based quantitative proteomics approach to study the protein expression profile alternations of the recurrence/metastasis of Huge HCC in different stage, and identified potentially interesting biomarkers for early distinguishing and predicting the recurrence/metastasis behaviors of H-HCC.

## Conclusions

We have applied the iTRAQ based quantitative proteomics approach to study the overall protein profile alternations in the early (within 6 months) and late (from 6–12 months) recurrence/metastasis of huge hepatocellular carcinoma after radical resection. The results proved different protein alternation profiles and different signaling pathways involving in the early and late recurrence/metastasis of H-HCC. Meanwhile, we identified and further verified S100A12 and AMACR might be considered as potentially interesting prognostic markers for predicting the early recurrence/metastasis of H-HCC after hepatectomy.

## Methods

### Sample collection

H-HCC patients who had undergone surgery at the liver center of the First Affiliated Hospital of Fujian Medical University of China from April 2008 to December 2010 were evaluated for inclusion in the current study. The absence of intrahepatic recurrence/metastasis in the residual liver was monitored by ultrasonography (US), computer tomography (CT) scan and angiography. CT scan was also carried out to rule out distant metastasis, including lung, brain and bone. The H-HCC patients were monitored for recurrence/metastasis every one month in the first year after surgery and every 3 months after 1 year post operation, including serum AFP, US and CT scan. Enrollment Eligibility Criteria: (1) The patient was diagnosed with HCC by post-operative pathological examination; (2) Pre-operative serum HBs Ag positive, but HBc Ab negative; (3) Subject to the standard radical resection [68]: no distal metastasis was revealed in both pre-and intra-operative examination; no lesion was found in the rest of the liver during intra-operative ultrasonic scan; no visible cancer embolus in the hepatic portal vein or primary venous branch; no cancer cell was found in the incisal margin at the post-operative pathological examination; no recurrent/metastatic lesion was found at the ultrasonic and CT scan during the return visit after 2 months of surgery; (4) the elevated pre-operative serum AFP should decline to the normal level after 2 months post operation; (5) The patient did not undergo any other intervention or therapies before surgery.

The tissues of patients were divided into 3 groups according to the time of recurrence/metastasis after operation: the patients who had recurrence/metastasis within 6 months after operation (R/M_≤6Months_ group, n = 20); the patients whose recurrence/metastasis occurred between 6 and 12 months after operation (R/M_6-12months_ group, n = 20); the patients who had no recurrence/metastasis within 2 years of operation (NR/M group, n = 20). Fresh tissues were collected at the time of surgery from patients with HBV associated H-HCC for liquid nitrogen preservation after washing, and part of the tissues were formalin embedded and stored for immunohistochemistry. The project was approved for the using of human biopsy by the Institution Review Board of the First Affiliated Hospital of Fujian Medical University. The written consent was received from all participants in this study.

### Protein preparation and iTRAQ labeling

**Figure 5:**
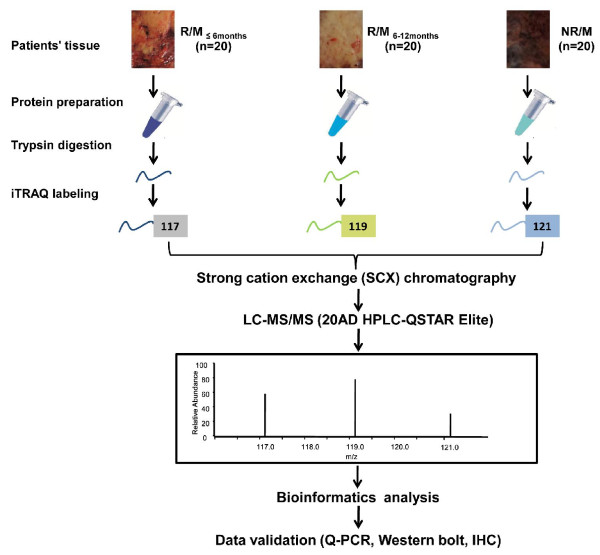
**Outline of the iTRAQ based quantitative proteomic strategy.** iTRAQ labeling was carried out by using tumor tissues from the patients who had recurrence/metastasis within 6 months after operation (R/M_≤6months_ group, n = 20); the patients whose recurrence/metastasis occurred between 6 and 12 months after operation (R/M_6-12months_ group, n = 20); and the patients who had no recurrence/metastasis within 2 years of operation (NR/M group, n = 20). Samples were digested by trypsin, and the peptides from R/M_≤6months_, R/M_6-12months_, and NR/M were labeled by iTRAQ reagents 117, 119 and 121, respectively. After labeling, peptides from all three samples were combined and fractionated by SCX chromatography. Each fraction was then analyzed by LC-MS/MS on a QSTAR Elite mass spectrometers. The identified potential interesting targets were further validated by Q-PCR, Western bolt and immunohistochemistry (IHC).

Ten milligrams of each liver tissue were lysed in the protein extraction buffer (150 mM NaCl, 10 mM Tris, 5 mM EDTA, 1% Triton X-100, 5% glycerol, and 0.1% SDS, pH 7.2) after smash in liquid nitrogen, and then incubated at 4°C for 30 min. After centrifugation at 12,000 rpm at 4°C for 30 min, the supernatant was collected, and the protein concentration was determined according to the BCA protocol (Beyotime, China). Samples were further normalized based on protein concentration. The iTRAQ labeling was performed according to the manufacturer’s protocol (AB SCIEX, USA). Briefly, 100 μg proteins of each group were precipitated with cold acetone for 1 hour at −20°C, and then re-suspended in 20 μl dissolution buffer. After protein reduction and alkylation followed by overnight digestion with trypsin, the peptides were labeled with the iTRAQ regents for 1 hour at room temperature. The iTRAQ regents 117, 119 and 121 were used to label the peptides from R/M_≤6Months_ group, R/M_6-12months_ group and NR/M group respectively. Then the samples were mixed with equal amounts, and desalted with the Sep-Pak Vac C_18_ cartridges and dried in a vacuum centrifuge. The workflow of our study was presented in Figure 5.

### 2DLC-MS/MS analysis

The mixed peptides were separated by strong cation exchange (SCX) chromatography using a polysulfoethanyl column (2.1 mm × 100 mm, 5 μm, 200 Å, The Nest Group, USA) at a flow rate of 200 μL/min for 60 min with a gradient of 0–80% Buffer B (10 mM KH_2_PO_4_ in 25% acetonitrile, 350 mM KCl, pH 2.6) in Buffer A (10 mM KH_2_PO_4_ in 25% acetonitrile, pH 2.6) on a 20 AD HPLC system (Shimadzu, Japan), and a total of 20 SCX fractions were collected.

The mixed peptides (desalted with a PepMap C_18_ cartridge) were further separated by nano-HPLC (20 AD, Shimadzu, Japan) on the secondary RP analytical column (ZORBAX 300SB-C_18_ column, 150 mm × 100 μm, 5 μm, 300 Å, USA). Peptides were subsequently eluted using the following gradient conditions with phase B (95% ACN with 0.1% formic acid): 5-35% B (0–90 min), 35–80% B (90–95 min), 80–5% B (100–105 min) and 5–0% B (105-120 min); the flow rate was maintained at 300 μL/min. Electrospray voltage of 2.3 kV versus the inlet of the mass spectrometer was used.

A hybrid quadrupole time-of-flight mass spectrometer (QStar hybrid LC/MS/MS Q-TOF, AB SCIEX, USA) was operated in data-dependent mode to switch automatically between MS and MS/MS acquisition. MS spectra were acquired across the mass range of 400–1800 m/z in high resolution mode using 250 ms accumulation time per spectrum. Tandem mass spectral scanned from 100–2000 m/z in high sensitivity mode with collision induced dissociation (CID). The four most intense precursors were selected for fragmentation per cycle with dynamic exclusion time of 9 s.

### Data analysis

The MS/MS data was searched against UniProtKB/ Swiss-Prot FASTA (it was released October 15, 2011 and consists of 20238 human sequences). Protein identification and iTRAQ quantitation were performed with ProteinPilot software (Version 4.0, AB SCIEX, USA). The user-defined search parameters included iTRAQ labeling at N-terminus and lysine residues, cysteine modification by methyl methanethiosulfonate (MMTS) and digestion by trypsin. For iTRAQ quantitation, the peptide was automatically selected with the Pro Group algorithm to calculate the reporter peak area, error factor (EF) and p value. A decoy database search strategy was adopted to estimate the FDR for peptide identification. In our study, a strict unused confidence cutoff >1.3 was used for protein identification; proteins with at least two peptides (confidence >95%) were used for quantification. The results were then exported into Microsoft Excel for manual data interpretation. The proteins were considered to be differentially expressed if their iTRAQ ratios were >2 or <0.5 in the R/M_≤6Months_ group and R/M_6-12months_ group relative to the NR/M group (P < 0.05) [69].

### Functional analysis

The biological function of all the identified proteins was analyzed in the online Gene Ontology (GO) Term mapper tool (http://go.princeton.edu/cgi-bin/GOTermMapper). The function and pathway annotations of identified proteins were analyzed by Ingenuity Pathways Analysis (IPA) software (version 7.5), which is based on the Ingenuity Pathways database. The involved signaling pathways and networks were ranked in term of the enrichment of the differentially expressed proteins.

### Immunoblotting

The tissue samples from patients were further taking for immunoblotting. The tissue samples were lysed in extraction buffer (150 mM NaCl, 10 mM Tris, 5 mM EDTA, 1% Triton X-100, 5% glycerol, and 0.1% SDS, pH 7.2), then were centrifuged at 22,000 rpm, 4°C for 30 min. The supernatants were collected, and the protein concentration was determined according to BCA protocol. Equal amount of proteins from each patient were applied for the immunoblotting experiments. 40 μg proteins per patients were analyzed on 12.5% SDS-PAGE, and transferred on PVDF membrane (transfer buffer: 25 mM Tris, 192 mM glycine, 20% methanol, pH8.3) by the Bio-Rad Semidry apparatus with a constant current of 300 mA for 1 hour or 30 min according to the size of the protein. Afterwards, the membrane was blocked in blocking buffer (1 × PBS, 0.5% Tween-20 with 5% nonfat milk) for 2 hours, and then further blotted with the primary antibody (monoclonal anti-S100A12 antibody, 1:500 dilution, Abcam; monoclonal anti-AMACR antibody, 1:1000 dilution, Abcam; monoclonal anti-β-actin antibody, 1:1000 dilution, Santa Cruz) in 4°C overnight and HRP conjugated secondary antibody in room temperature for 2 hrs. Afterwards, the membrane was incubated with Chemi-luminescent Detection Reagent (Pierce) for 5 min, and then was exposed to X-ray film. All of the above mentioned experiments were independently repeated 3 times.

### Immunohistochemistry of Liver sections

The expression profile of the protein S100A12 and AMACR in liver section of the patients was investigated by the two-step Envision™ plus staining technique. The paraffin-embedded liver sections were de-waxed and re-hydrated through incubating in EDTA (1 mM) solution with 3 minutes for high-pressure antigen retrieval. Afterwards, the liver section was incubated with the primary antibody (mouse monoclonal anti-human S100A12 antibody, 1:500 dilution, Abcam; mouse monoclonal anti-human AMACR antibody, 1:1000 dilution, Abcam) at 4°C overnight, and then washed by PBS buffer for 3 times (5 min of each) at room temperature. It further followed by incubating with HRP-conjugated secondary antibodies (Fuzhou Maxim Biotech Inc, China) at room temperature for another 1 hour. Finally, the liver sections were subjected to DAB coloration and hematoxylin re-staining. The results were independently assessed by two pathologists double-blindly.

### Q-PCR analysis

Trizol homogenization buffer (Takara Inc.) was added into the tissue samples to extract the total RNAs, which were then reversely transcribed into cDNAs (Reverse transcription was conducted according to the manufacture’s protocol from Takara). The β-actin was taken as the internal reference gene. The obtained cDNAs were used as the template for the Q-PCR analysis of the S100A12 and AMACR genes in a total reaction system of 50 μL (including 1 μL of the cDNA template, 1 μL of the upstream primer, 1 μL of the down stream primer, 25 μL of the SYBR green super mix and 22 μL of the RNase free dH_2_O). The reaction conditions were as follows: 94°C, 3 min, 1 cycle; followed by 40 cycles of 95°C 15 s, 62°C 15 s (S100A12) or 60°C 15 s (AMACR), 60°C 40 s. The relative fluorescence intensity from 55°C to 95°C (0.5°C increments every 10 sec) was collected to plot the melting curve, so as to survey the formation of primer dimmers and the non-specific amplification. The relative gene expression was calculated according to the Livak method (2^-ΔΔCt^). The experiments were repeated 3 times independently. The sequence information of the primers was as follows:

S100A12, forward primer 5′-ATTAGGCTGGGAAGATGACAAA-3′, and the reverse primer 5′-GCTTCAGCTCACCCTTAGAGAG-3′;

AMACR, forward primer 5′-ATTTGGCTTTGTCAGGTGTTCT-3′, and the reverse primer 5′- GCGGTCAAAAAGAGCCATTAT-3′;

β-actin, forward primer 5′-CCACTGGCATCGTGATGGAC-3′, and the reverse primer 5′- GCGGATGTCCACGTCACACT-3′.

## Abbreviations

2DLC-MS/MS: Multidimensional chromatography tandem mass spectrometry; GO: Gene Ontology; KEGG: Kyoto Encyclopedia of Genes and Genomes.

## Competing interests

The authors declare no competing interests.

## Authors′ contributions

XH and YZ performed the iTRAQ experiments; XX and XL performed the data analysis and paper writing; YZ, JZ and BX performed the clinical sample collection; XH, YZ, YG and ZC performed the validation experiments; XL, AH and JL designed the experiment. All authors read and approved the final manuscript.

## Supplementary Material

Additional file 1 Tables of Identified Proteins. Click here for file

Additional file 2 Supporting Information. Click here for file
