# Supplementary material for: Quantitative proteomics analysis of early recurrence/metastasis of huge hepatocellular carcinoma following radical resection
Source: Proteome Sci. 2014 May 1;12:22. doi: 10.1186/1477-5956-12-22 (PMC4023177; doi:10.1186/1477-5956-12-22)
Supplement: Additional file 2 — Supporting Information. [file 1477-5956-12-22-S2.docx]

**Supporting Information**

**Figure S1**


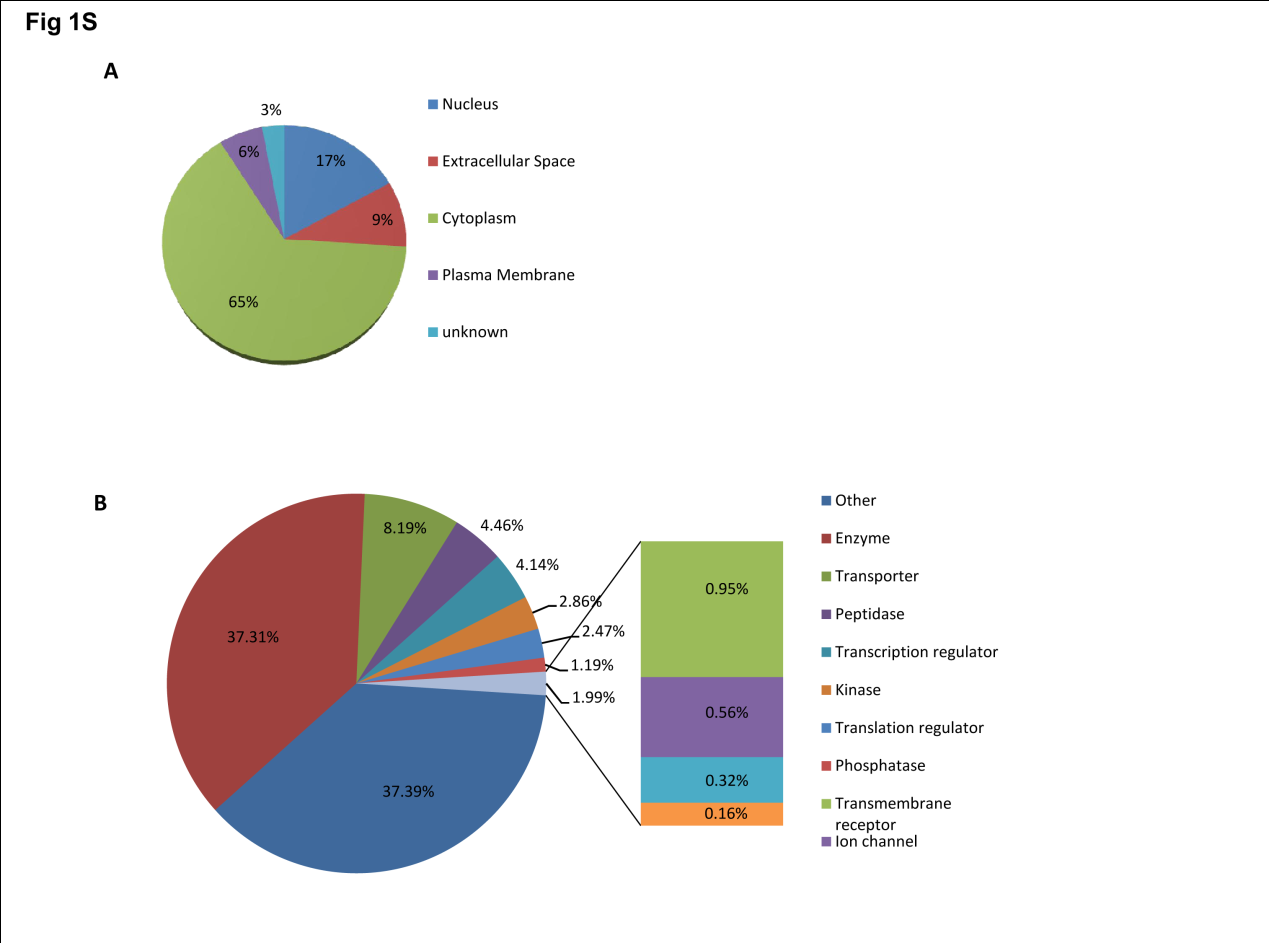


Fig. S1 Localization annotation of proteins identified from R/M_≤6months_ and R/M_6-12months_. (A) Sub-cellular localization of identified proteins. (B) Molecular type of the identified proteins.

**Figure S2**


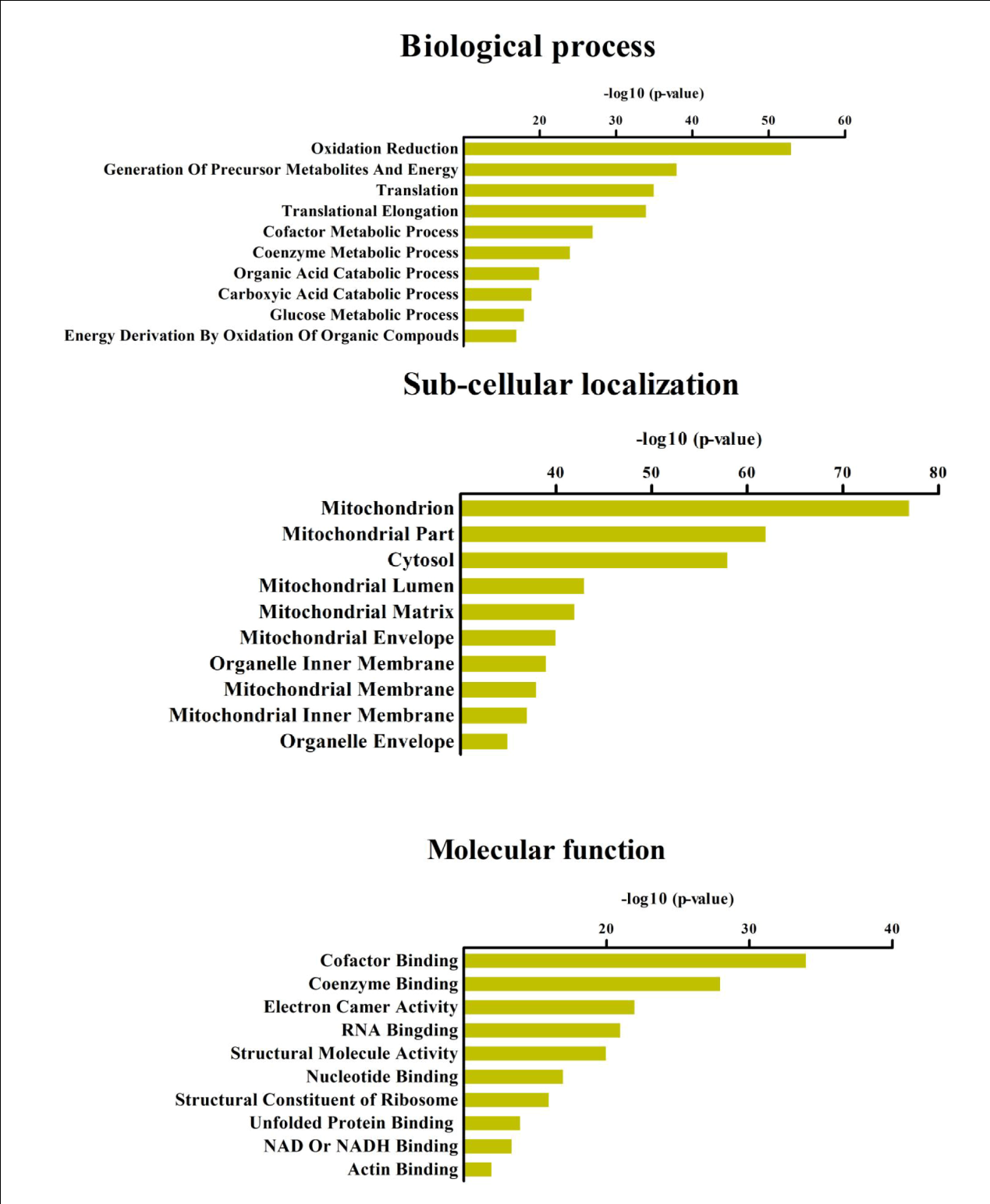


Fig. S2 Gene ontology (Go) function analysis of all the identified proteins; the top 10 were presented. (A) Involved biological process of all identified proteins. (B) Sub-cellular localization of all identified proteins. (C) Molecular function of all identified proteins.

**Figure S3**

**
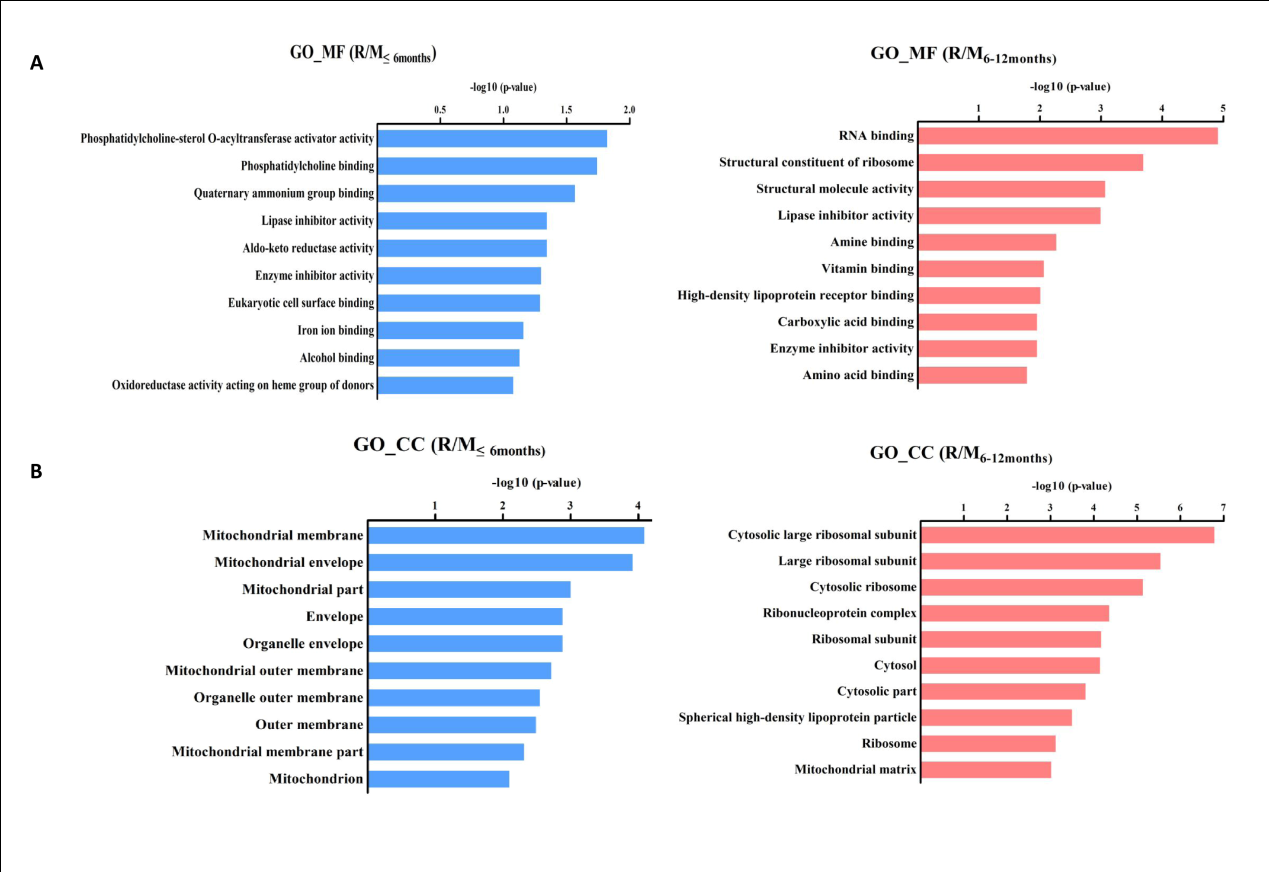
**

Fig. S3 Go function analysis of the differentially expressed proteins in R/M_≤6months_ and R/M_6-12months_ group, the top 10 were presented. (A) Cell component of the differentially expressed proteins in R/M_≤6months_ and R/M_6-12months_ group. (B) Molecular function of the differentially expressed proteins in R/M_≤6months_ and R/M_6-12months_ group.
